# ^90^Y-Labeled Anti-ROBO1 Monoclonal Antibody Exhibits Antitumor Activity against Small Cell Lung Cancer Xenografts

**DOI:** 10.1371/journal.pone.0125468

**Published:** 2015-05-27

**Authors:** Kentaro Fujiwara, Keitaro Koyama, Kosuke Suga, Masako Ikemura, Yasutaka Saito, Akihiro Hino, Hiroko Iwanari, Osamu Kusano-Arai, Kenichi Mitsui, Hiroyuki Kasahara, Masashi Fukayama, Tatsuhiko Kodama, Takao Hamakubo, Toshimitsu Momose

**Affiliations:** 1 Department of Radiology, Graduate School of Medicine, The University of Tokyo, Bunkyo-ku, Tokyo, Japan; 2 SANKYO LABO SERVICE Co., Ltd., Edogawaku, Tokyo, Japan; 3 Department of Pathology, Graduate School of Medicine, The University of Tokyo, Bunkyo-ku, Tokyo, Japan; 4 FUJIFILM RI Pharma Co., Ltd., SAMMU-CITY, CHIBA, Japan; 5 Department of Quantitative Biology and Medicine, Research Center for Advanced Science and Technology, The University of Tokyo, Meguro-ku, Tokyo, Japan; 6 Department of Systems Biology and Medicine, Research Center for Advanced Science and Technology, The University of Tokyo, Meguro-ku, Tokyo, Japan; Duke University Medical Center, UNITED STATES

## Abstract

**Introduction:**

ROBO1 is a membrane protein that contributes to tumor metastasis and angiogenesis. We previously reported that ^90^Y-labeled anti-ROBO1 monoclonal antibody (^90^Y-anti-ROBO1 IgG) showed an antitumor effect against ROBO1-positive tumors. In this study, we performed a biodistribution study and radioimmunotherapy (RIT) against ROBO1-positive small cell lung cancer (SCLC) models.

**Methods:**

For the biodistribution study, ^111^In-labeled anti-ROBO1 monoclonal antibody (^111^In-anti-ROBO1 IgG) was injected into ROBO1-positive SCLC xenograft mice via the tail vein. To evaluate antitumor effects, an RIT study was performed, and SCLC xenograft mice were treated with ^90^Y-anti-ROBO1 IgG. Tumor volume and body weight were periodically measured throughout the experiments. The tumors and organs of mice were then collected, and a pathological analysis was carried out.

**Results:**

As a result of the biodistribution study, we observed tumor uptake of ^111^In-anti-ROBO1 IgG. The liver, kidney, spleen, and lung showed comparably high accumulation of ^111^In-labeled anti-ROBO1. In the RIT study, ^90^Y-anti-ROBO1 IgG significantly reduced tumor volume compared with baseline. Pathological analyses of tumors revealed coagulation necrosis and fatal degeneration of tumor cells, significant reduction in the number of Ki-67-positive cells, and an increase in the number of apoptotic cells. A transient reduction of hematopoietic cells was observed in the spleen, sternum, and femur.

**Conclusions:**

These results suggest that RIT with ^90^Y-anti-ROBO1 IgG is a promising treatment for ROBO1-positive SCLC.

## Introduction

Small cell lung cancer (SCLC) constitutes approximately 15% of all lung cancer cases and exhibits a high growth rate and early development of widespread metastases. Accordingly, most patients with SCLC have hematogenous metastases at initial diagnosis[1, 2]. Since SCLC is highly sensitive to initial chemotherapy and radiotherapy, these methods are commonly used to treat SCLC[1, 3]; however, the overall prognosis of SCLC patients is poor. The 2-year survival rate for patients with limited-stage disease is 20–40%, while the 2-year survival rate for patients with advanced disease is only 5% because advanced disease is typically treated with chemotherapy alone[2]. Therefore, the development of more effective SCLC treatments for both limited-stage disease and extensive-stage disease (primary lesions and metastasis) is required.

Radioimmunotherapy (RIT) is a type of radiotherapy using radiolabeled tumor-specific monoclonal antibody[4–6]. Although RIT treatments Zevalin and Bexxar have been successfully used to treat relapsed or refractory non-Hodgkin's lymphomas, the success of RIT as treatment for solid tumors has been limited. This is due to both the radioresistance of solid tumors and the inability to deliver a sufficient dose to bulky tumors without causing bone-marrow toxicity[4, 7]. Recently, Yoshida et al. reported that RIT shows the significant therapeutic efficacy for SCLC xenografts[8]. The clinical trial of anti-CEA radioimmunotherapy in SCLC has been initiated[3]. SCLC has high radiosensitivity, although it is likely to become metastatic. RIT can attack both primary and metastatic cancer because RIT agent is delivered to all parts of the body via blood circulation. These suggest that RIT has the potential to become an effective treatment for SCLC.

The human homologue of the *Drosophila* Roundabout gene, *ROBO1*, is a membrane protein and a receptor of slit2 [9]. Slit2-ROBO1 interactions mediate repulsive cues in axons and growth cones during neural development[9, 10]. It has been reported that ROBO1 contributes to both tumor metastasis and angiogenesis[11–13]. We previously conducted an RIT study in hepatocellular carcinoma (HCC) targeting the ROBO1 antigen[14]. Administration of ^90^Y-labeled anti-ROBO1 IgG (^90^Y-anti-ROBO1 IgG) showed significant antitumor effects, such as tumor growth suppression in ROBO1-positive HCC xenografts. However, tumor shrinkage was not observed, perhaps owing to the radioresistance of HCC. Therefore, we determined that a more radiosensitive tumor than HCC would be an appropriate therapeutic target for ^90^Y-anti-ROBO1 IgG. SCLC has high radiosensitivity compared with HCC. Xian et al. reported the expression of ROBO1 in human SCLC cell line NCI-H69[15]. Therefore, we selected the SCLC model using NCI-H69 cell line as a more effective therapeutic target for use in the present study.

In this study, we investigated the pharmacokinetics of an ^111^In-labeled anti-ROBO1 IgG (^111^In-anti-ROBO1 IgG) in a biodistribution study using SCLC xenograft mice. In addition, we performed RIT using a ^90^Y-anti-ROBO1 IgG, and the antitumor effect and damage to organs were evaluated by pathological analysis.

## Materials and Methods

### Cell culture and animal models

The ROBO1-positive SCLC cell line NCI-H69 (ATCC HTB-119) was obtained from American Type Culture Collection[15]. Male BALB/c nude mice (5 weeks of age) were purchased from Clea Japan, Inc. (Tokyo, Japan). NCI-H69 cells were cultured in RPMI 1640 medium containing 10% (v/v) fetal bovine serum at 37°C in a humidified atmosphere with 5% CO_2_. For the NCI-H69 tumor-bearing animal models, NCI-H69 cells (2 × 10^6^), in a volume of 200 μl, were inoculated subcutaneously into the right flanks of male BALB/c nude mice. Mice were then analyzed 4 weeks after inoculation. Mice were euthanized by blood removal under isoflurane anesthesia. All animal studies were approved by the Animal Care Committee of the University of Tokyo (approved number: P10-017).

### Antibodies

Monoclonal antibody against human ROBO1 was generated as previously described[16]. Anti-ROBO1 IgG is a murine IgG2b. It recognize human ROBO1 and does not cross-react with murine ROBO1. For immunohistochemical (IHC) analysis, we used anti-ROBO1 IgG A7241A (A7241A)[16]. For flow cytometry, the biodistribution study and the RIT study, anti-ROBO1 IgG B5209B (B5209B) was used[14]. A negative control, IgG 3423 (Institute of Immunology, Tokyo, Japan), was used in flow cytometry. This monoclonal antibody specifically reacts with hepatitis B surface antigen[17]. Moreover, it does not react with human membrane proteins.

### Flow cytometry and IHC

The specificity of B5209B for the ROBO1 antigen was evaluated using flow cytometry. NCI-H69 cells were incubated with B5209B or a negative control IgG 3423 for 1 h at a concentration of 0.3 μg/ml in dilution buffer (1% BSA, 0.1 mM EDTA in PBS). Simultaneously, to demonstrate the specificity of B5209B, blocking experiments were conducted by adding soluble ROBO1 at a concentration of 100 μg/ml. The cells were then washed twice with dilution buffer and reacted with R-Phycoerythrin-conjugated anti-mouse IgG (Jackson ImmunoResearch Laboratories, West Grove, PA, USA) diluted to 1:200 with dilution buffer. Finally, the cells were washed twice with dilution buffer and analyzed by flow cytometry (GUAVA EasyCyteTM Plus System; Millipore, Billerica, MA, USA).

The expression of ROBO1 in NCI-H69 xenografts was evaluated using IHC. NCI-H69 tumor tissue samples were fixed in 4% paraformaldehyde overnight at 4°C. They were embedded in paraffin, and 3–5 μm-thick sections were obtained. Tissue sections were deparaffinized and rehydrated. Antigen retrieval was performed in 10 mM Tris-1 mM EDTA buffer (pH 9.0) in a pressure cooker for 20 min. Endogenous peroxidase was quenched using 0.3% hydrogen peroxide-methanol for 30 min, and then the specimens were blocked with 5% normal goat serum for 1 hour. The specimens were incubated with A7241A (10 μg/ml) overnight at 4°C and then treated with a secondary antibody (Simple stain MAX-PO; Nichirei, Tokyo, Japan) at room temperature for 30 min. To visualize peroxidase activity, 0.2 mg/ml 3,3’-diaminobenzidine (DAB; Dojindo Laboratories, Kumamoto, Japan) was used as the substrate in 0.05 M Tris-HCl buffer (pH 7.6) containing 0.01% H_2_O_2_. Nuclear staining was performed with hematoxylin.

### Radiolabelling

We purchased 1, 4, 7, 10-tetraazacyclododecane-1, 4, 7, 10-tetraacetic acid (DOTA) from Macrocyclics (Dallas, TX, USA), ^111^InCl_3_ was obtained from Nordion, Inc. (Vancouver, Canada), and ^90^YCl_3_ was obtained from Eckert and Ziegler (Braunschweig, Germany).

B5209B was conjugated with DOTA and radiolabeled with ^111^In and ^90^Y as previously described[14]. We reported that the immunoreactive fraction of radiolabeled B5209B does not impair conjugation with intact B5209B[14].

### Biodistribution study

NCI-H69 xenograft (300.7 ± 125 mm^3^) mice were randomly divided into 5 groups (n = 3 per group). Each mouse was injected with 40 μCi of ^111^In-B5209B (80 μg) via the tail vein. The mice were euthanized at 6, 24, 48, 72, and 144 h after injection. Blood, heart, lung, liver, kidney, spleen, stomach, intestine, muscle, femoral bone, sternum, and tumor were collected, weighed, and measured for radioactivity. The percentage of injected dose per gram of tissue (% ID/g) was calculated for each organ.

The tumor absorbed dose for ^90^Y-B5209B was estimated from the biodistribution data of ^111^In-B5209B. Tumor accumulation at various tome points were plotted against time, from which the area under the curve (AUC) was calculated. Tumor absorbed dose was estimated based on a MIRD-style mouse dosimetry model[18]. The absorbed dose to organs in a 70-kg reference man was estimated from our biodistrubution data using MIRDOSE3 computer program[19].

### Therapeutic study

NCI-H69 xenograft mice were randomly divided into 2 groups (n = 3 per group). Mice were injected via the tail vein with saline or 200 μCi of ^90^Y-B5209B (70 μg). Tumor volumes for the control and ^90^Y-B5209B groups were 310.5 ± 128.6 mm^3^ and 273.5 ± 153.6 mm^3^, respectively. The tumor volume and body weight were measured twice a week until 4 weeks after injection. Tumor volume was calculated using the following formula: 0.5 × (shortest diameter)^2^ × (longest diameter). Tumor growth (%) was calculated using the formula: (tumor volume at each time point)/(tumor volume on day 0) × 100. Mice were euthanized when tumor size was > 1,500 mm^3^ or body weight decreased by > 20% from original weight.

### Pathological study

NCI-H69 xenograft mice were randomly divided into 5 groups (n = 3 per group). Mice were injected via the tail vein with 200 μCi of ^90^Y-B5209B. The mice were euthanized at days 0, 7, 14, 21, and 28 after injection, and then tissue specimens of NCI-H69 tumor, liver, kidney, lung, heart, intestine, spleen, pancreas, sternum, and femur were obtained from the mice.

All samples were fixed in 4% paraformaldehyde overnight at 4°C. They were embedded in paraffin, and 3–5-μm thick sections were obtained. The sternum and femur were decalcified before embedding in paraffin. Slide sections, excluding the femoral bone and sternum, were deparaffinized, dehydrated, and stained with hematoxylin and eosin. Femoral bone and sternum sections were stained with Giemsa solution.

Terminal deoxynucleotidyl transferase-mediated dUTP nick end labeling (TUNEL) and Ki-67 staining were performed on NCI-H69 sections to investigate the status of tumor apoptosis and cell proliferation, respectively.

Apoptosis was detected using ApopTag Plus Peroxidase In Situ Apoptosis Detection Kit (Chemicon International, INC, Billerica, MA), per the manufacturer’s instructions.

Ki-67 staining was performed as previously described [14].

Apoptotic and proliferative cells were quantified by determining the percentage of TUNEL- and Ki-67-positive cells, respectively, as previously described [14].

### Statistical analysis

Data were expressed as the mean ± standard deviation. Means were compared using the Student’s *t*-test. P values of < 0.05 were considered statistically significant.

## Results

### The specificity of B5209B to ROBO1 antigen

**Fig 1:**
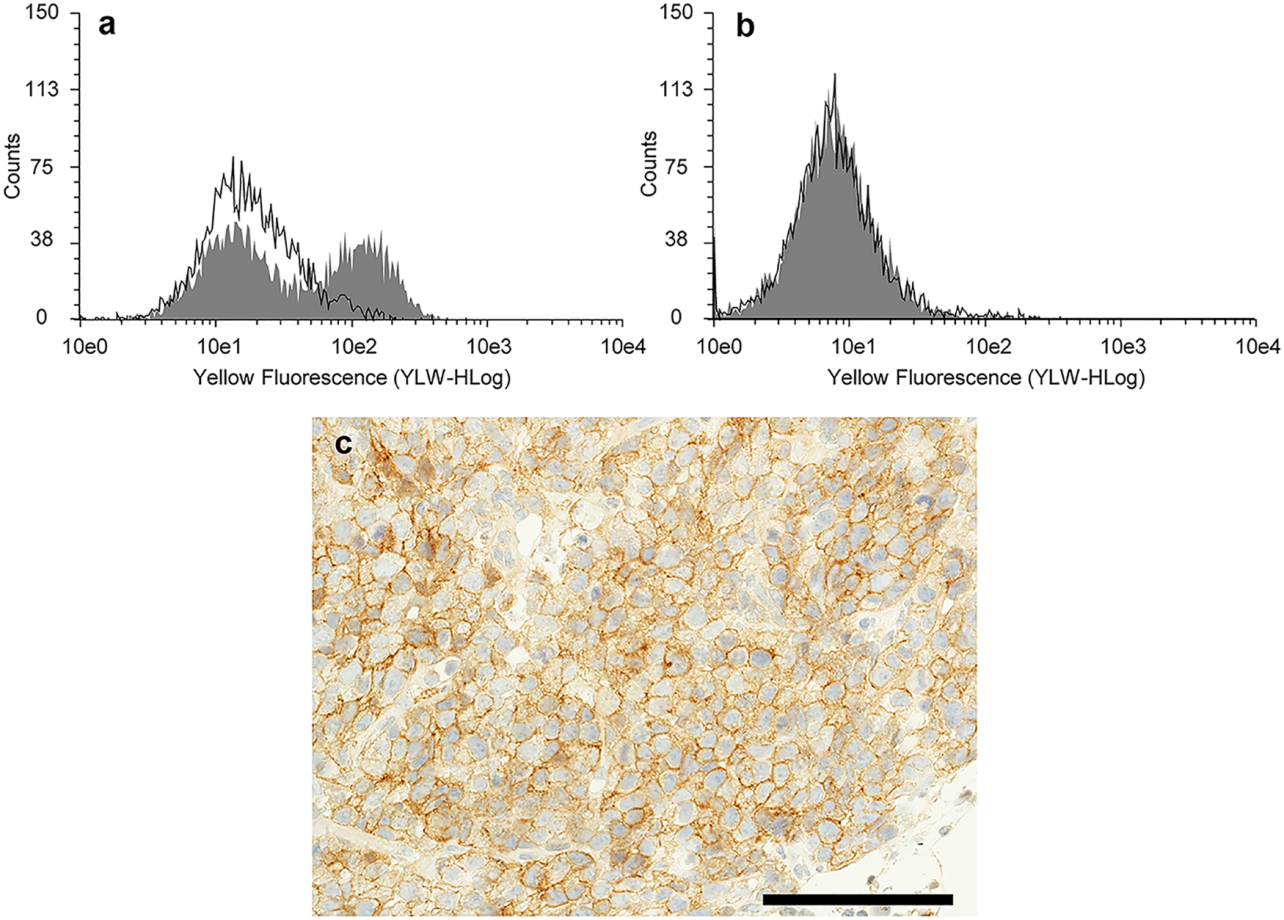
The specificity of B5209B and the expression of ROBO1 in NCI-H69 xenografts. (a) Flow cytometric analysis with B5209B (filled histogram) in NCI-H69 cell line. Open histograms indicate the blocking experiments which used soluble ROBO1. (b) Flow cytometric analysis with IgG 3423 (filled histogram). Open histograms indicate the blocking experiments which used soluble ROBO1. (c) Immunohistochemical analysis of ROBO1 with A7241A in NCI-H69 xenograft.(scale bar = 100 μm).

NCI-H69 cells exhibited mean fluorescence intensities of 35.29 and 7.80 with B5209B and negative control IgG 3423, respectively (Fig 1a and 1b). Binding of B5209B was inhibited by adding soluble ROBO1.

### ROBO1 expression in NCI-H69 xenografts

The expression of ROBO1 in NCI-H69 xenografts was confirmed by IHC using A7241A. Staining with A7241A was observed on the cell membranes, as ROBO1 is expressed on the cell surface (Fig 1c).

### Biodistribution study

**Fig 2:**
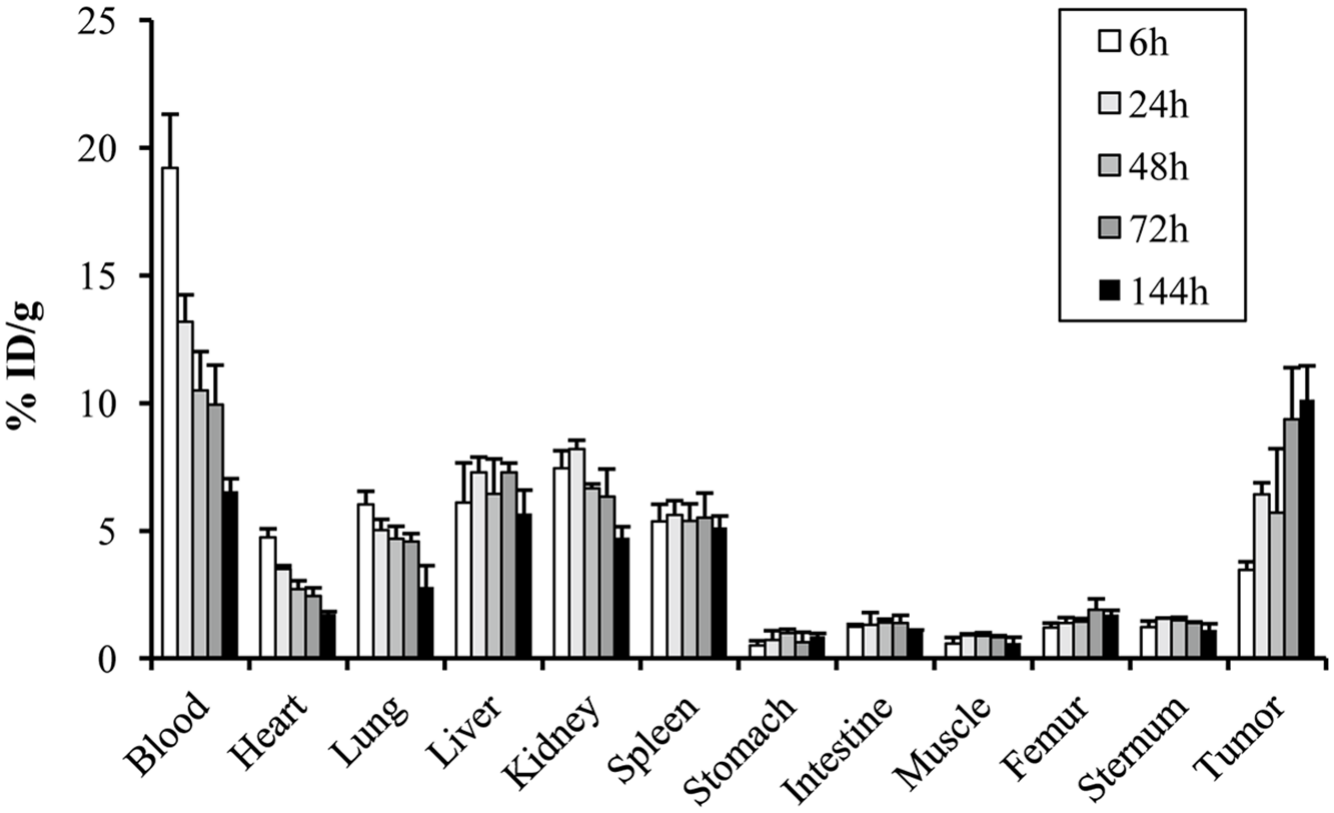
Biodistribution of ^111^In-B5209B in NCI-H69 xenograft mice. Results represent the calculated percentages of the injected dose per gram of tissue (% ID/g).

The biodistribution study using ^111^In-B5209B was carried out using NCI-H69 xenograft mice (Fig 2). The tumor uptake of ^111^In-B5209B was 3.46 ± 0.3%, 6.42 ± 0.5%, 5.70 ± 2.5%, 9.37 ± 2.0%, and 10.1 ± 1.3% ID/g at 6, 24, 48, 72, and 144 h after injection, respectively. The maximal tumor uptake of ^111^In-B5209B occurred at 144 h after injection. High retention of tracer in the blood was observed with 19.2 ± 2.1% ID/g at 6 h, but this decreased to 6.55 ± 0.5% ID/g at 144 h. ^111^In-B5209B showed high uptake in the liver, kidney, and spleen with 5.78 ± 0.9%, 4.73 ± 0.4%, and 5.13 ± 0.4% ID/g at 144 h, respectively.

The dose absorbed by tumors treated with 200 μCi of ^90^Y-B5209B was estimated to be 10.5 Gy. The doses absorbed by the liver, kidney, spleen, and bone marrow were estimated from our biodistribution data to be 2.1 mGy/MBq, 3.8 mGy/MBq, 1.3 mGy/MBq, and 0.4 mGy/MBq, respectively, in a 70-kg male reference subject for ^90^Y-B5209B.

### RIT study

**Fig 3:**
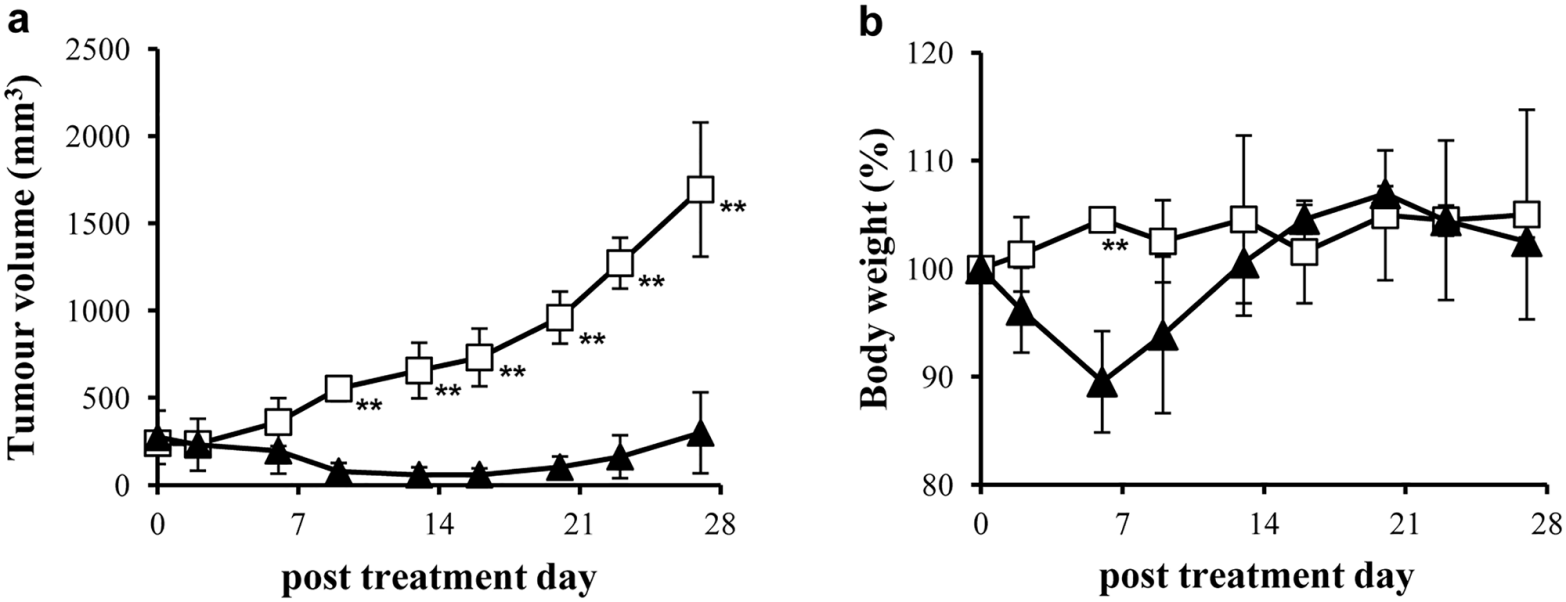
Effect of ^90^Y-B5209B on tumor growth and body weight. (a) tumor growth curve, (b) Mean body weight of NCI-H69 xenograft mice (open square, saline; black triangles, ^90^Y-B5209B). ** p < 0.01 (student's t-test).

The therapeutic efficacy of ^90^Y-B5209B was investigated in NCI-H69 xenograft mice (Fig 3a). The tumors in the saline group showed rapid growth. One mouse in the saline group was euthanized at day 20 because the tumor volume reached 1,500 mm^3^. In the ^90^Y-B5209B group, tumor volume decreased to 19.3 ± 6.1% on day 13 compared with the original volume on day 0. Tumor volume showed significant differences between groups from day 9 onward (p < 0.01), although no significant difference was observed on day 0, 2, or 6. Tumor regrowth was observed on day 20.

Animal body weight was measured after injection (Fig 3b). Although the average body weight decreased to 89.5 ± 4.7% of the original value in the ^90^Y-B5209B group, this change was transient. The mice in the saline group showed no significant reduction in body weight. On day 6, body weight differed significantly between the saline group and the ^90^Y-B5209B group (p < 0.01). On day 9, body weight showed no significant difference between the two groups.

### Pathological analysis

**Fig 4:**
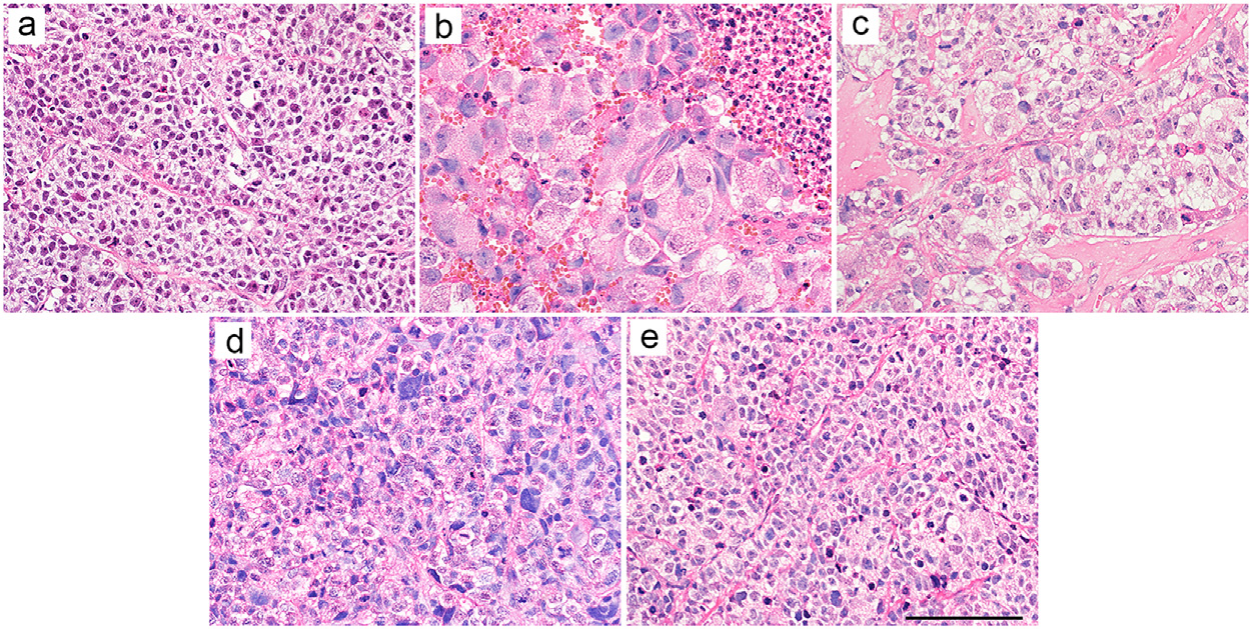
Histological analysis of tumors. (a) NCI-H69 tumor on day 0, original magnification × 400; (b) NCI-H69 tumor on day 7, original magnification × 400; (c) NCI-H69 tumor on day 0, original magnification × 400; (d) NCI-H69 tumor on day 0, original magnification × 400; (e) NCI-H69 tumor on day 0, original magnification × 400. Scale bar = 100 μm.

We then evaluated the antitumor effect by pathological analysis. On day 0, tumor cells showed solid growth associated with hyalinized fibrous stroma (Fig 4a).

On day 7, we observed massive coagulation necrosis, apoptotic cells, and cell degeneration, defined by cell body swelling, cell nuclear swelling, and chromatin compaction (Fig 4b).

On day 14, in addition to coagulation necrosis and cell degeneration, we also observed histiocyte aggregation and fibrosis in necrotic regions (Fig 4c).

On day 21, necrotic regions were not observed, while histiocyte aggregation and fibrosis were observed (Fig 4d). Although the number of degenerating cells decreased compared with day 14, we still observed many degenerating cells.

On day 28, few degenerating cells were observed (Fig 4e). The characteristics of the cell body and nucleus, such as shape and size, were not altered and appeared to be the same as those observed on day 0.

**Fig 5:**
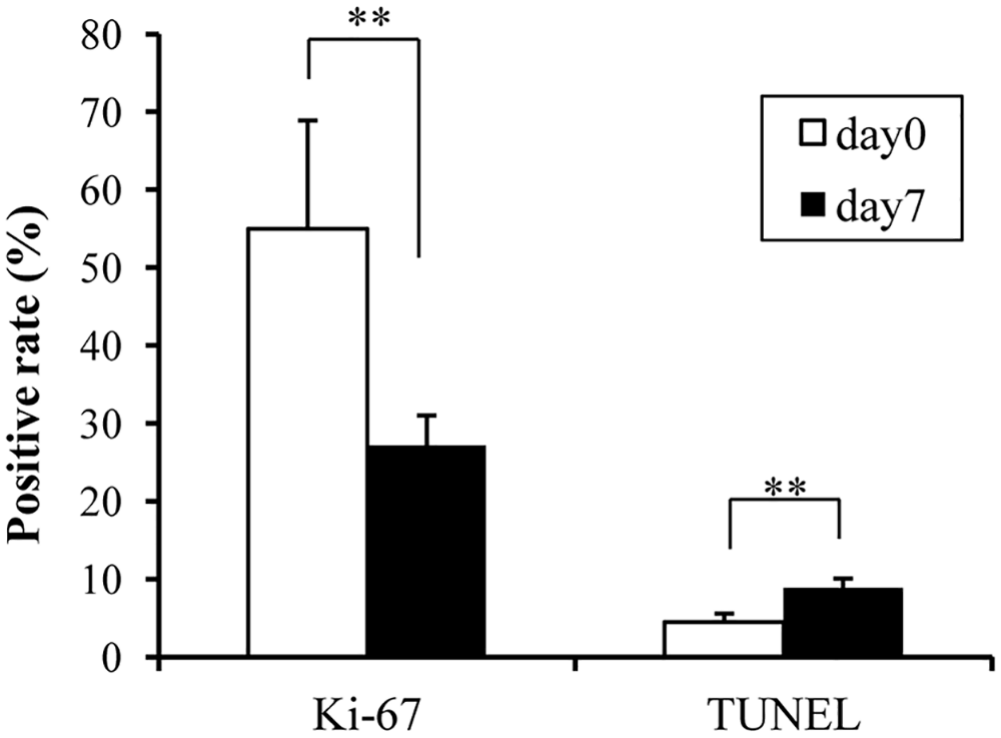
Quantitative analysis of cell proliferation and apoptosis. Ki-67: ratio of Ki-67-positive cells; TUNEL: ratio of TUNEL-positive cells. *p < 0.01 (students' t-test).

S1 Fig shows the Ki-67 staining and TUNEL staining. The percentage of Ki-67-positive cells on day 7 decreased significantly (27.2 ± 3.4%) compared with day 0 (55.0 ± 13.9%; p < 0.01; Fig 5). The percentage of TUNEL-positive cells on day 7 increased significantly (8.83 ± 1.2%) compared with day 0 (4.46 ± 1.1%; p < 0.01; Fig 5).

No apparent pathological changes were observed in the normal organs, excluding the spleen, sternum, femur, and liver.

**Fig 6:**
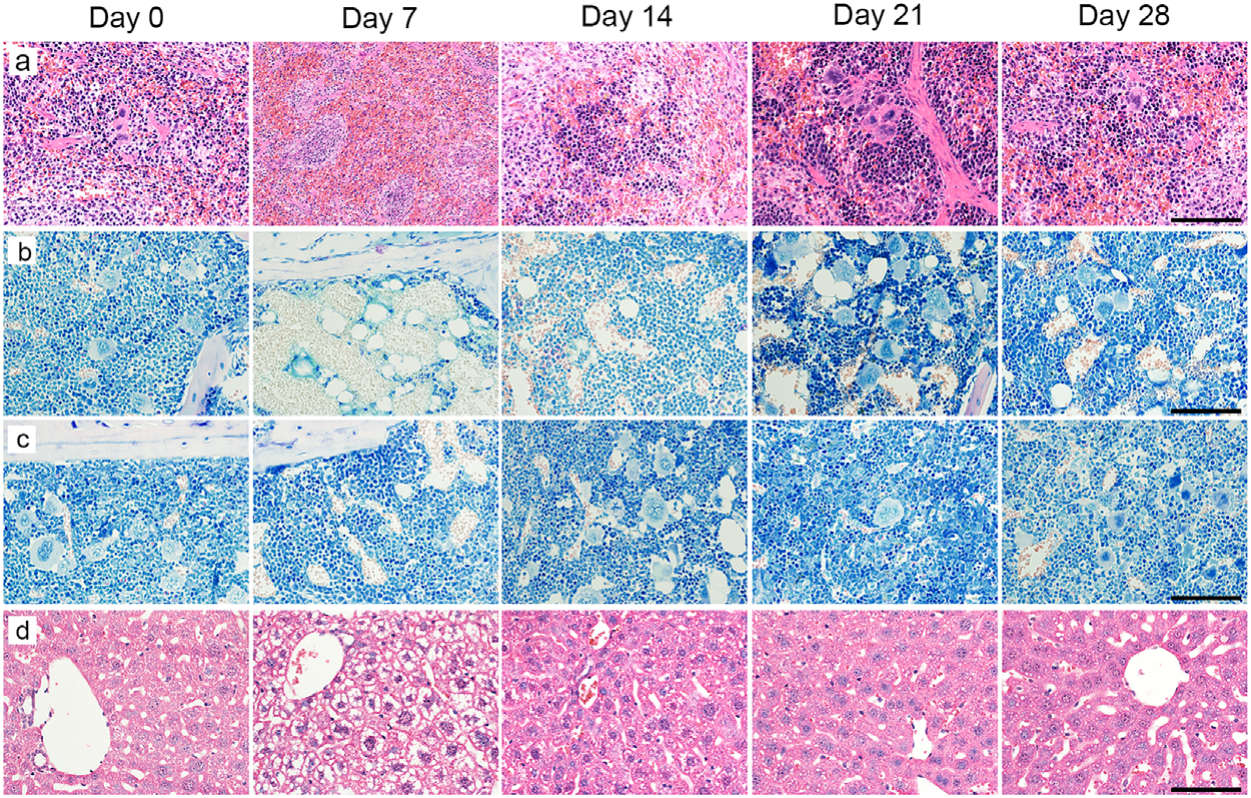
Histological analysis of the spleen, sternum, femur, and liver. (a) Spleen, original magnification × 400, scale bar = 100 μm. (b) Sternum, original magnification × 400, scale bar = 100 μm. (c) Femur, original magnification × 400, scale bar = 100 μm. (d) Liver, original magnification × 400, scale bar = 100 μm.

On day 7, the spleen showed a decrease in numbers of both hematopoietic cells in the red pulp and lymphocytes in the white pulp (Fig 6a). On day 14, regenerative islands, composed of a mix of erythroblast and granulocytic cells, were observed in the red pulp of the spleen. In the white pulp of the spleen, the cell density of lymphocytes was in the same range as that observed on day 7. On day 21, hematopoietic cells, including erythroblasts, granulocytes, and megakaryocytes, were significantly increased in the red pulp of the spleen. Moreover, a small increase of lymphocytes was observed in the white pulp. On day 28, the cell density of hematopoietic cells appeared to be the same as that observed on day 0 in the red pulp of the spleen, and lymphocytes were significantly increased in the white pulp.

We then performed Giemsa staining of the sternum and femur (Fig 6b and 6c). On day 7, the sternum bone marrow showed a remarkable decrease in numbers of hematopoietic cells. Mature granulocytes made up the vast majority of the remaining hematopoietic cells. In the femoral bone marrow, we observed a small decrease in the number of megakaryocytes, while there was no apparent decrease in numbers of erythroblasts and granulocytes. The damage to the femoral bone marrow was mild compared to that observed in the sternum bone marrow. On day 14, regenerative islands were observed in the sternum bone marrow, and few megakaryocytes were present. The femoral bone marrow exhibited an increase in megakaryocytes, and the cell density of hematopoietic cells appeared to be the same as that observed on day 0. On day 21, the sternum bone marrow showed a significant increase in the number of hematopoietic cells. On day 28, the cell density of hematopoietic cells in the sternum bone marrow appeared to be the same as that observed on day 0.

On day 7, ballooning of hepatocyte numbers was observed in the liver (Fig 6d), while this was not seen on day 14. In addition, the characteristics of hepatocytes appeared to be the same as those observed on day 0.

## Discussion

This study is the first to demonstrate that ^111^In-B5209B accumulates within NCI-H69 xenografts, and ^90^Y-B5209B has significant antitumor effects against NCI-H69 xenografts.

To evaluate the specificity of B5209B for ROBO1, we performed a blocking study using flow cytometry. The binding of B5209B was inhibited by adding soluble ROBO1, although the same treatment does not inhibit the binding of IgG 3423. Therefore, B5209B specifically recognizes the ROBO1 antigen.

We used ^111^In-anti-ROBO1 as a biodistribution surrogate to predict in vivo behaviors of ^90^Y-anti-ROBO1; this was because it has been reported that ^111^In and ^90^Y-labeled antibodies, proteins and peptides are biologically equivalent, with respect to their uptake in tumors and other major organs [20].

We confirmed that B5209B has specificity for the ROBO1 antigen. In addition, the uptake of ^111^In-B5209B in NCI-H69 increased gradually over time, and the maximum uptake by tumors was 10.1 ± 1.3% ID/g at 144 h. Uptake of radiotracer to normal organs which do not express ROBO1, such as liver, kidney and spleen, showed relatively high accumulation. However, the uptake of each organ either decreased gradually over time or remained constant. These uptake behaviors differed from the uptake observed in tumors. Therefore, the results of our blocking and biodistribution study support that ^111^In-B5209B specifically targets ROBO1. It is expected that ^90^Y-B5209B would accumulate in NCI-H69 xenografts, because of similar binding affinity[14].

Although ^111^In-B5209B showed relatively high accumulation in the liver, kidney, and spleen, the binding specificity of ^111^In-B5209B cannot explain this high accumulation, because these organs do not express ROBO1[16]. This phenomenon may be explained by the presence of residual ^111^In-B5209B in the blood and Fcγ or FcRn receptor-mediated absorption by the endothelial cells in these organs[21, 22].

We injected 200 μCi of ^90^Y-B5209B into NCI-H69 xenograft mice, and the tumor volume of NCI-H69 xenografts decreased significantly after treatment with ^90^Y-B5209B. After ^90^Y-B5209B administration, we observed pathological changes in the tumor, including cell degeneration, coagulation necrosis, an increase in apoptotic cells, and a decrease of Ki-67-positive cells. These changes are common pathological changes in treated tumor tissues. Thus, these results suggest that ^90^Y-B5209B possesses significant antitumor effects when used in RIT.

Unfortunately, we observed tumor regrowth on day 20 in the ^90^Y-B5209B group. In the pathological study, decreased cell degeneration was observed from day 21 to day 28. There was no necrotic region on day 21, although histiocyte aggregation and fibrosis were observed at that time, and these are immune responses to necrotic cells. Histiocytes ingest necrotic cells, and fibroblasts repair that region. In addition, the morphological characteristics of cell bodies and nuclei on day 28 appeared to be the same as those observed on day 0. These results suggest that the antitumor effects of RIT waned at day 21. To achieve complete remission or prolonged survival using ^90^Y-B5209B, enhancement or extension of these antitumor effects is necessary. Combination therapy with RIT, chemotherapy and poly ADP ribose polymerase inhibitor results in prolonged survival compared with each therapy alone[23, 24]; therefore, combination therapy has a potential to deliver more effective antitumor activity. The usage of long-half life nuclide may achieve improvement of therapeutic effects. ^90^Y is a useful therapeutic β^-^ emitter that has high maximum energy (2.3 MeV) and a long range (maximum tissue penetration 11 mm)[25]. However, its half-life is relatively short (2.7 days). Compared with ^90^Y, ^177^Lu has lower maximum energy (0.5 MeV) and a shorter range (maximum tissue penetration 1.6mm), but it has a longer half-life (6.7 days)[26]. Thus, use of ^177^Lu may extend the period of antitumor effects and limit the damage to organs because of its short range. In addition, it has been reported that ^177^Lu RIT is more effective than with ^90^Y RIT in the treatment of small lesions (< 1,000 mm^3^) [27, 28]. ^177^Lu RIT may be more effective for the relatively small tumors which we used in this study.

Yoshida et al. reported on the effect of radioimmunotherapy using the ^90^Y-anti-c-kit antibody for SCLC[8]. The ^90^Y-anti-c-kit antibody achieved a complete therapeutic response for SCLC xenografts. The two studies differed in several ways, such as the targeted protein, the antibody used, and tumor volume. The tumor volume used in our study (273.5 ± 153.6 mm^3^) was more than 50-fold greater than the tumor volume used in2 their study (4.4 ± 3.2 mm^3^). The difference in tumor volume may explain the disparity in therapeutic effect observed between the two studies, because tumor volume is the main factor in determining treatment outcome in radiotherapy[29]. Moreover, the availability of RIT treatment depends on the presence or absence of target protein expression. Therefore, a wider variety of target proteins for RIT is preferable. ^90^Y-B5209B has value as a therapeutic candidate for the treatment of ROBO1-positive SCLC because c-kit is not always expressed in SCLC.

We also observed body weight loss, decreased hematopoietic cells and ballooning of hepatocytes. These conditions were transient, indicating that 200 μCi of ^90^Y-B5209B was under the maximum tolerated dose in NCI-H69 xenograft mice. It is necessary to perform the dose-escalation study for the determination of a lethal dose and optimal therapeutic dose. Consideration of the absorbed dose to the normal organs is important in determining the therapeutic radiation dose in patients. In this study, we estimated the absorbed dose to organs in a 70-kg reference man from our biodistribution data. The doses absorbed by the liver, kidney, spleen and bone marrow were 2.1 mGy/MBq, 3.8 mGy/MBq, 1.3 mGy/MBq, and 0.4 mGy/MBq, respectively. In a clinical study of ^90^Y-Zevalin, the acceptable absorbed doses to normal organs and the red marrow were below 20 Gy and 3 Gy, respectively[30]. The maximum tolerated doses, calculated from doses absorbed by the liver, kidney, spleen, and bone marrow, were 9.5 GBq, 5.3 GBq, 15.4 GBq, and 7.5 GBq, respectively. Hence, the estimated maximum therapeutic dose of ^90^Y-B5209B is 5.3 GBq. However, it is reported that biodistribution of this radiolabeled antibody shows a discrepancy between animal models and patients[31]. Moreover, B5209B reacts to human ROBO1 but not to murine ROBO1. It is necessary to perform a biodistribution study with ^111^In-B5209B in patients to estimate accurate dosimetry.

A difference in the degree of damage between the sternum and femur was observed. However, the accumulation of ^111^In-B5209B has no difference between the two in our biodistribution study. These suggest that the accumulation of ^90^Y-B5209B to these organs is not a main factor of the difference in the degree of damage between the two. We previously explained that this difference may be due to the interval disparity from blood pool and high accumulation areas such as tumor, liver and spleen[14]. This suggests that the degree of side effect in RIT has the potential to change depending on a metastatic position. Therefore, it is necessary to identify focal positions before the injection of ^90^Y-labeled RIT agent for the estimation of side effect.

In the spleen, we observed regenerative islands composed of erythroblast and granulocytic cells on days 14 and 21, along with a significant increase in the number of hematopoietic cells, including megakaryocytes. These results suggested that the regeneration of hematopoietic tissues began on day 14–20, and the damage to hematopoietic tissues was transient with this treatment. The increase of megakaryocytes was observed after the increase in erythroblasts and granulocytes. In addition, we observed a decrease in megakaryocytes, without erythroblasts and granulocytes, in the femur. Kashiwakura et al. reported that colony-forming unit megakaryocytes are more radiosensitive than other myeloid progenitor cells[32]. Therefore, the high radiosensitivity of colony-forming unit megakaryocytes may explain these phenomena.

On day 7, we observed a ballooning of hepatocyte in the liver. Because ^111^In-B5209B showed high accumulation to the liver in the biodistribution study, ^90^Y-B5209B is hypothesized to have similar biodistribution. Therefore, this cell degeneration was likely induced by exposure to ^90^Y-B5209B in the liver. However, the ballooning of hepatocyte was reversible, as this was not observed on day 14. This suggests that the damage to liver was transient, and no fatal effect was observed using this injected dose.

As a limitation of this study, although we observed significant antitumor effects and transient damage to organs, an optimal dosage of ^90^Y-B5209B and a contribution to survival benefit are uncertain. We did not performed a dose escalation study because our objective was not optimization of injection dose but an evaluation of the antitumor effect on SCLC xenografts, and determination of whether ^90^Y-B5209B causes damage to organs in terms of pathology. The dose of 200 μCi was determined by reference to a previous study, the dose of 180 μCi of ^90^Y-B5209B was not fatal, and the dose of 200 μCi of the other antibody provided maximal antitumor efficacy without animal mortality[14, 33]. In addition, SCLC has a higher radiosensitivity than HCC although the uptake of ^111^In-B5209B to SCLC xenograft was low compared with that in HCC xenograft[14]. Therefore, we expected that the injection of 200 μCi of ^90^Y-B5209B provides the results which can achieve our objective in this study. A dose escalation study and long-term observation are required to optimize the injection dose and to evaluate the contribution to survival benefit.

## Conclusions

We evaluated a radiolabeled anti-ROBO1 IgG as a possible candidate for RIT of SCLC. Based on the biodistribution study, ^111^In-B5209B showed high accumulation to NCI-H69 xenografts, representative of human SCLC. Moreover, ^90^Y-B5209B showed antitumor activity towards NCI-H69 xenografts, which was confirmed by a pathological study. In conclusion, our data suggest that ^90^Y-B5209B, which targets ROBO1, is a promising treatment for ROBO1-positive SCLC.

## Supporting Information

S1 Fig Immunohistochemistry and TUNEL stain for NCI-H69. (a) Ki-67 stain of NCI-H69 tumor on day 0, original magnification × 400; (b) Ki-67 stain of NCI-H69 tumor on day 7, original magnification × 400; (c) TUNEL stain of NCI-H69 tumor on day 0, original magnification × 400; (a) TUNEL stain of NCI-H69 tumor on day 7, original magnification × 400. Scale bar = 100 μm.
(TIF)
