# Supplementary figures and images for: 90Y-Labeled Anti-ROBO1 Monoclonal Antibody Exhibits Antitumor Activity against Small Cell Lung Cancer Xenografts
Source: PLoS One. 2015 May 27;10(5):e0125468. doi: 10.1371/journal.pone.0125468 (PMC4446100; doi:10.1371/journal.pone.0125468)

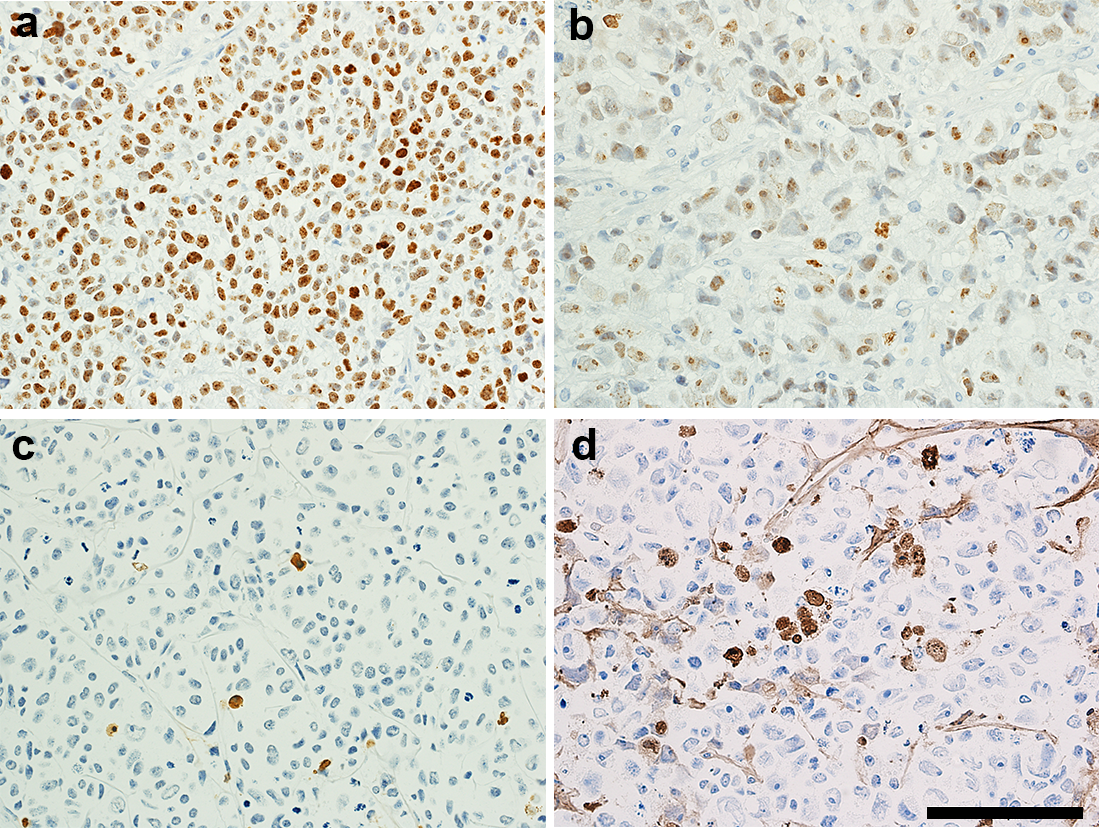

Supplement: S1 Fig — (a) Ki-67 stain of NCI-H69 tumor on day 0, original magnification × 400; (b) Ki-67 stain of NCI-H69 tumor on day 7, original magnification × 400; (c) TUNEL stain of NCI-H69 tumor on day 0, original magnification × 400; (a) TUNEL stain of NCI-H69 tumor on day 7, original magnification × 400. Scale bar = 100 μm. (TIF) [file pone.0125468.s001.tif]
